# Risk of the hydrogen economy for atmospheric methane

**DOI:** 10.1038/s41467-022-35419-7

**Published:** 2022-12-13

**Authors:** Matteo B. Bertagni, Stephen W. Pacala, Fabien Paulot, Amilcare Porporato

**Affiliations:** 1 The High Meadows Environmental Institute, Princeton University, Guyot Hall, Princeton, 08544 NJ USA; 2 Department of Ecology and Evolutionary Biology, Princeton University, Guyot Hall, Princeton, 08544 NJ USA; 3 Geophysical Fluid Dynamics Laboratory, National Oceanic and Atmospheric Administration, 201 Forrestal Rd, Princeton, 08540 NJ USA; 4 Department of Civil and Environmental Engineering, Princeton University, Guyot Hall, Princeton, 08544 NJ USA

**Keywords:** Atmospheric chemistry, Environmental impact, Natural gas, Hydrogen fuel

## Abstract

Hydrogen (H_2_) is expected to play a crucial role in reducing greenhouse gas emissions. However, hydrogen losses to the atmosphere impact atmospheric chemistry, including positive feedback on methane (CH_4_), the second most important greenhouse gas. Here we investigate through a minimalist model the response of atmospheric methane to fossil fuel displacement by hydrogen. We find that CH_4_ concentration may increase or decrease depending on the amount of hydrogen lost to the atmosphere and the methane emissions associated with hydrogen production. Green H_2_ can mitigate atmospheric methane if hydrogen losses throughout the value chain are below 9 ± 3%. Blue H_2_ can reduce methane emissions only if methane losses are below 1%. We address and discuss the main uncertainties in our results and the implications for the decarbonization of the energy sector.

## Abstract (web summary)

H_2_ has the potential to become the green, low-carbon fuel of the future. However, hydrogen emissions impact atmospheric methane (CH_4_). Bertagni et al. investigate the fate of atmospheric CH_4_ in scenarios of H_2_ economy.

## Introduction

Commitments to reach net-zero carbon emissions have drawn renewed attention to hydrogen (H_2_) as a low-carbon energy carrier^1,2^. Currently, H_2_ is mostly used as an industrial feedstock, and its global production has a high carbon footprint because it relies almost entirely (≈95%) on fossil fuels^1^. However, many technologies to produce H_2_ with a lower carbon footprint are available^1^. Among these, low-carbon H_2_ can be produced from water electrolysis powered by renewable energy (green H_2_) or from methane reforming coupled with carbon capture and storage (blue H_2_). H_2_ fuel may be especially important to decarbonize energy and transport sectors where direct electrification is complicated, like heavy industry, heavy-duty road transport, shipping, and aviation^1^. H_2_ is also being considered for storing renewable energy^1^. As a result of this potential, countries accounting for more than a third of the world’s population have developed national strategies for large-scale H_2_ production^1,2^.

Even if a more hydrogen-based economy would reduce CO_2_ emissions and improve air quality^3^, it would also increase the H_2_ emissions into the atmosphere. The H_2_ molecule is very small and difficult to contain, so it is still largely unknown how much H_2_ will leak in future value chains. H_2_ emissions will also occur due to venting, purging, and incomplete combustion^4–6^. This potential increase in H_2_ emissions has received relatively little attention to date because H_2_ is neither a pollutant nor a greenhouse gas (GHG). However, it has been long known^7–10^ that H_2_ emissions may exert a significant indirect radiative forcing by perturbing the concentration of other GHG gases in the atmosphere. This indirect GHG effect of H_2_ calls for a detailed scrutiny of the global H_2_ budget and the environmental consequences of its perturbation^11,12^.

H_2_ is the second most abundant reactive trace gas in the atmosphere, after methane, with an average concentration of around 530 ppb_v_^13^. H_2_ sources include both direct emissions (≈45% of total sources) and production in the troposphere from the oxidation of volatile organic compounds (≈25%) and methane (≈30%)^11,14^. The main H_2_ sinks are the uptake by soil bacteria (70–80% of total tropospheric removal) and the atmospheric reaction with the radical OH (20–30%), which is responsible for the indirect GHG effect of H_2_. H_2_’s reaction with the OH radical tends to increase tropospheric methane (CH_4_) and ozone (O_3_), which are two potent greenhouse gases. It also increases stratospheric water vapor, which is associated with stratospheric cooling and tropospheric warming^8,15^. Recent global climate models have estimated that hydrogen has an indirect radiative forcing of around 1.3^14^–1.8^16^ 10^−4^ W m^−2^ ppb${}_{{{{{{{{\rm{v}}}}}}}}}^{-1}$, and a global warming potential (GWP) that lies in the range 11 ± 5 for a 100-year time horizon^16^. Hence, H_2_ emissions are far from being climate neutral, and their largest impact is related to the perturbation of atmospheric CH_4_^14,16^, the second most important anthropogenic GHG.

**Fig. 1:**
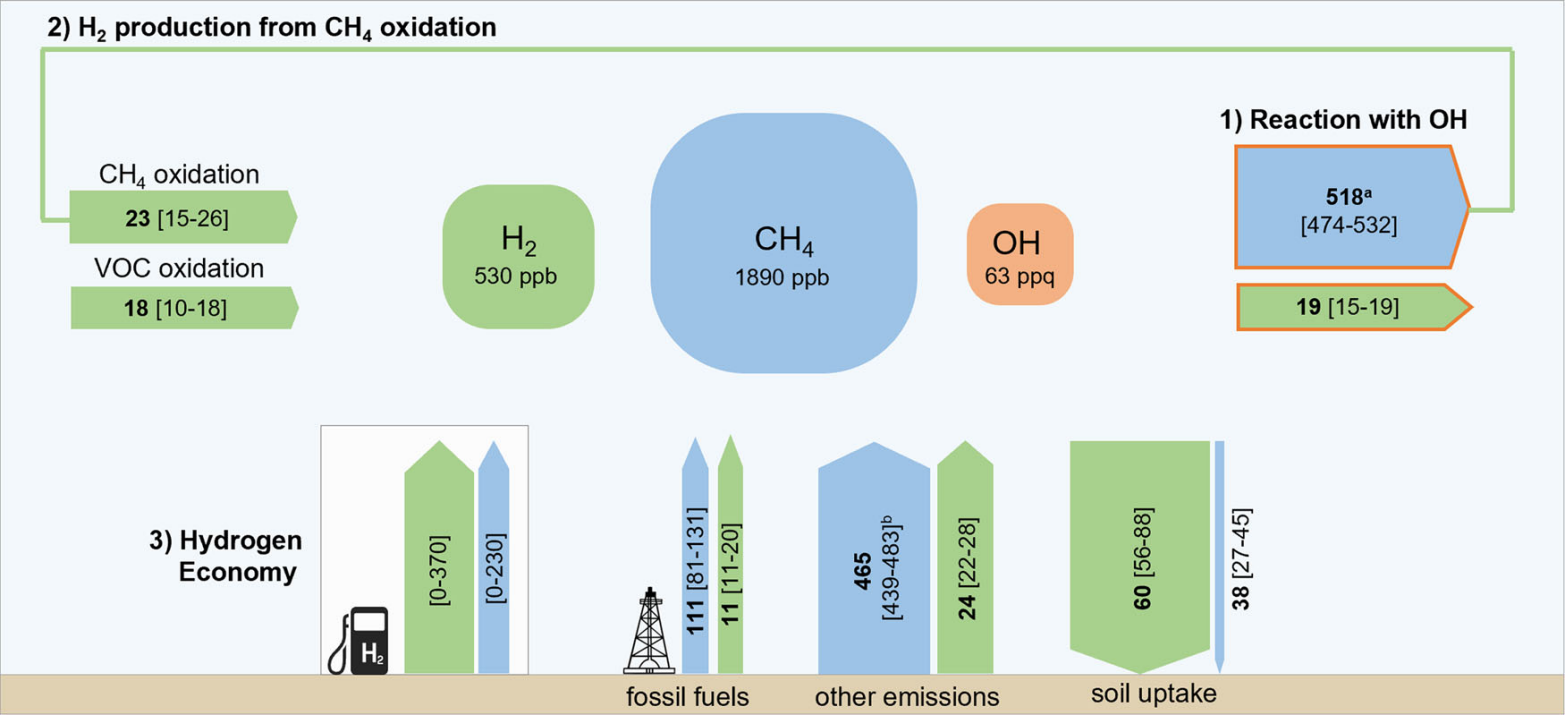
Tangled hydrogen (H_2_) and methane (CH_4_) budgets. Sketch of H_2_ and CH_4_ tropospheric budgets and their interconnections: (1) the competition for OH; (2) the production of H_2_ from CH_4_ oxidation; (3) the potential emissions [minimum-maximum] due to a more hydrogen-based energy system. Flux estimates (Tg/year) are from refs. 11,18. Arrows are scaled with mass flux intensity, CH_4_ scale being 10 times narrower than H_2_ scale. On a per-mole basis, H_2_ consumes only around 3 times less OH than CH_4_. ppq = part per quadrillon (10^−15^). ^*a*^ top-down estimate including also minor atmospheric sinks (<10%). ^*b*^ range obtained as a difference between total and fossil fuel emissions^18^.

The tropospheric budgets of H_2_ and CH_4_ are deeply interconnected (Fig. 1). First, the removal of both gases from the atmosphere is controlled by their reaction with OH, which is the dominant sink (≈90%) for atmospheric methane^17,18^. An increase in the concentration of tropospheric H_2_ may reduce the availability of OH, consequently weakening CH_4_’s removal and increasing CH_4_’s lifetime and abundance^14,19^. Second, methane is a primary precursor of hydrogen. Namely, CH_4_ oxidation results in the production of formaldehyde, whose photolysis produces H_2_. Firn-air records suggest that the increase in H_2_ over the 20th century can be largely explained by the increase in CH_4_ concentration^20^.

Additionally, H_2_ and CH_4_ are linked at the industrial level. Around 60% of global H_2_ production is currently produced from steam methane reforming (gray H_2_) and is responsible for 6% of global natural gas use^1^. In the next decade, steam methane reforming coupled with carbon capture and storage will likely remain the dominant technology for large-scale H_2_ production (blue H_2_), since facilities for H_2_ production from renewable sources (green H_2_) will require time to become operational and economically favorable^2^.

Since CH_4_ is the second-largest contributor to atmospheric warming since the beginning of the industrial era and there are global efforts to mitigate its atmospheric levels^21^, it is crucial to quantify the response of atmospheric CH_4_ to increasing H_2_ production.

We analyze this problem through a simple atmospheric model that captures the interaction between H_2_ and CH_4_ (“Methods”). The investigation of the transient dynamics (“Methods”) shows that any H_2_ emissions pulse to the atmosphere leads to a small transient growth of atmospheric CH_4_ whose effects last for several decades. In the next sections, we focus on how the equilibrium concentrations of tropospheric H_2_ and CH_4_ would respond to scenarios of continuous emissions from an energy system where part of the fossil fuel energy share is replaced by green or blue H_2_. The analysis emphasizes how atmospheric CH_4_ could either decrease or increase, mainly depending on the H_2_ production pathway and the amount of H_2_ lost to the atmosphere. The latter is defined through the hydrogen emission intensity (HEI), namely the percentage of H_2_ produced that is lost to the atmosphere. Specifically, we find a critical HEI above which the CH_4_ atmospheric burden rises despite the lower fossil fuel use. We assess the critical factors and the main uncertainties in the quantification of this critical HEI. We finally discuss how our results can help better inform policymakers regarding the trade-off associated with different scenarios of hydrogen production and use.

## Results

### Emission scenarios

Here we investigate how the tropospheric burdens of methane and hydrogen would be affected by the transition to a more hydrogen-based energy system, wherein hydrogen replaces part of the current fossil fuel energy (≈490 ExJ in 2019^22^). To achieve this goal, we estimate the CH_4_ and H_2_ source changes, ${{\Delta }}{S}_{{{{{{{{{\rm{CH}}}}}}}}}_{4}}$ and ${{\Delta }}{S}_{{{{{{{{{\rm{H}}}}}}}}}_{2}}$, where Δ indicates the difference to the current tropospheric conditions (“Methods”). This fossil fuel displacement reduces both CH_4_ and H_2_ sources (Fig. 1). The rise in H_2_ production causes additional H_2_ emissions due to intentional (e.g., venting) and unintended (e.g., fugitive) losses, and possibly CH_4_ emissions associated with blue H_2_ production.

**Fig. 2:**
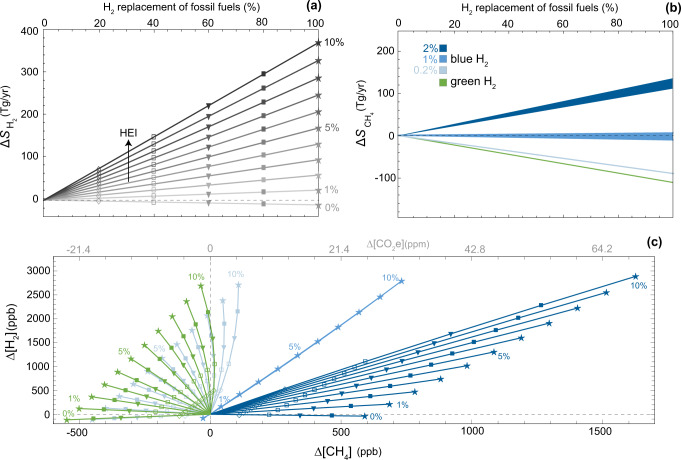
Hydrogen replacement of fossil fuels. **a** Changes in H_2_ sources (${{\Delta }}{S}_{{{{{{{{{\rm{H}}}}}}}}}_{2}}$) as a function of fossil fuel replacement for different hydrogen emission intensity (HEI). **b** Changes in CH_4_ sources (${{\Delta }}{S}_{{{{{{{{{\rm{CH}}}}}}}}}_{4}}$) as a function of fossil fuel replacement for different H_2_ production pathways. Methane leak rates associated with blue H_2_ production are 0.2, 1, and 2%. Bands for ${{\Delta }}{S}_{{{{{{{{{\rm{CH}}}}}}}}}_{4}}$ account for different amounts of blue H_2_ produced and lost. **c** Response of the tropospheric concentrations of H_2_ and CH_4_ for the emission scenarios of the previous panels. Symbols mark the different percentages of fossil fuel displacement. Only symbols for 100% fossil fuel replacement are reported for blue H_2_ with 1% CH_4_ leakage. Also reported is the difference in CO_2_ concentration (Δ[CO_2_e]) that would produce equivalent radiative forcing to the change in equilibrium CH_4_ (upper axis).

The change in H_2_ emissions can be estimated from the amount of hydrogen produced to substitute fossil fuels and the HEI, namely the percentage of H_2_ produced that is lost to the atmosphere. Losses can occur due to venting, purging, incomplete combustion and leaks across the hydrogen value chain. The HEI of the future global H_2_ value chain is very uncertain. Literature values range from 1 to 12%^4,9,23^, but the upper bound is unlikely to occur at large scales because it would be both unsafe and too expensive. Recent empirical estimates for specific H_2_ infrastructures suggest HEI’s ranging from 0.1 to 6.9%, critically depending on the pathway of hydrogen production and transport^6^. To account for these uncertainties and to explore a broad spectrum of possible scenarios, here we vary HEI from 0 to 10% of the total hydrogen produced (Fig. 2a). The lower and upper bounds of this range represent a perfectly sealed and a highly leaking global H_2_ value chain, respectively. With a perfectly sealed hydrogen value chain, H_2_ emissions would only decrease due to the lower fossil fuel use. On the contrary, a highly leaking H_2_ value chain, coupled with an envisioned penetration of H_2_ in the energy market, could increase hydrogen emissions up to several times the total current sources, which are around 80 Tg H_2_ yr^−1^.

The variation in CH_4_ emissions depends not only on the percentage of fossil fuel energy that is displaced by hydrogen, but also on the hydrogen production pathway. For green H_2_, i.e., hydrogen obtained from renewable sources, we scale CH_4_ emissions based on the reduced consumption of fossil fuels resulting from hydrogen usage (Fig. 2b). Estimates of current methane emissions associated with fossil fuel extraction and distribution are in the range 80–160 Tg CH_4_ yr^−1^^18,24,25^ and relatively equally distributed among coal, oil, and gas sectors^26^. Here we use the top-down estimate of 111 Tg/year^18^.

For blue H_2_, which is derived from steam methane reforming (SMR), the variation in CH_4_ sources not only accounts for the reduced consumption of fossil fuels but also for the methane emissions (venting, incomplete combustion, fugitive) associated with blue hydrogen production. These emissions depend on the amount of CH_4_ needed to produce H_2_, i.e., feedstock and energy requirements of the SMR process (“Methods”), and the CH_4_ leak rate. The precise average leak rate of the global natural gas supply chain remains uncertain. One of the reasons is that national inventories generally underestimate real emissions^27–30^. More detailed studies relying on field measurements in the United States and Canada estimate average leak rates around 2%^28–30^, with large spatial heterogeneity between different operators^31^. Although national inventories suggest that some countries, like Venezuela and Turkmenistan, have higher leak rates^26^, here we adopt 2% as the maximum global CH_4_ leak rate for our scenarios, because methane-mitigation efforts are likely to decrease future global leak rates^21^ and, more importantly, because not all hydrogen produced will be blue H_2_. In this regard, the scenario of blue H_2_ with a 2% CH_4_ leak rate can also be interpreted as a combination of equal production of green H_2_ and blue H_2_ with 4% CH_4_ leak rate. We use 0.2% as a lower bound for the CH_4_ leak rate, since this has been declared as the target of several energy companies for 2025^32^. 1% represents an intermediate scenario of blue H_2_ production.

Figure 2b shows the resulting CH_4_ emissions associated with green and blue H_2_ production with methane leak rates of 0.2, 1, and 2%. The different leak rates have a great impact on the methane emissions. Compared to the fossil fuel energy system, CH_4_ emissions are reduced in the blue H_2_ scenario with 0.2% methane losses, but largely increased in the blue H_2_ scenario with 2% methane losses. The fossil fuel displacement by blue H_2_ with 1% methane losses shows basically no net effect on the CH_4_ emissions.

**Fig. 3:**
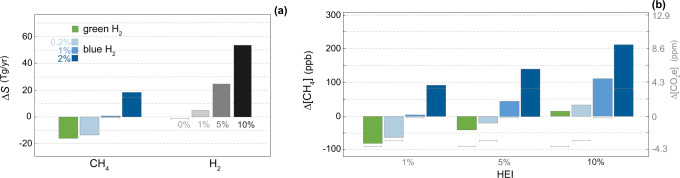
Methane response to increasing H_2_ production. **a** Changes in H_2_ and CH_4_ sources (Δ*S*) due to green and blue H_2_ production (≈500 Tg yr^−1^). HEI is the H_2_ emission intensity. Gray lines mark the case for HEI = 0%. Blue bars for ${{\Delta }}{S}_{{{{{{{{{\rm{CH}}}}}}}}}_{4}}$ are obtained with HEI = 10%. **b** Response of CH_4_ atmospheric concentration. The right axis shows the Δ[CO_2_e] that would produce equivalent radiative forcing to the change in equilibrium CH_4_.

As a specific case, we also investigate the H_2_ and CH_4_ emission changes associated with estimates of future hydrogen production in a set of net-zero scenarios. H_2_ production is expected to increase from current 90 Tg/year to 530–660 Tg/year in 2050^2,33,34^. We thus consider a 500 Tg/year rise in the global H_2_ production, which is energetically equivalent to about 15% of current fossil fuel energy. Figure 3a shows how, depending on the H_2_ production pathway and the different hydrogen and methane leak rates, the emission changes of these two gases can vary substantially.

### Tropospheric response

For the previous emission scenarios, we evaluate the changes in the equilibrium concentrations of tropospheric hydrogen and methane, namely Δ[H_2_] and Δ[CH_4_]. The timescales to equilibrium are dictated by the gas average lifetimes (“Methods”). The corresponding variations in steady state concentration of OH are reported in Supplementary Figs. 1 and 2.

The H_2_ economy causes a rise in tropospheric H_2_ as a result of the additional emissions (Fig. 2c). The intensity of this increase varies considerably as a function of the emissions of the hydrogen value chain. The concentration variation could go from less than 100 ppb_v_ to more than 2000 ppb in envisioned scenarios of the H_2_ economy, namely a +300% from the current H_2_ tropospheric level.

The response of atmospheric CH_4_ results from the combination of the methane emission change and the methane sink weakening due to the higher hydrogen emissions. To discriminate between the two mechanisms, it is useful to focus on the scenarios of fossil fuel displacement by green H_2_. In the case of a perfectly sealed green H_2_ value chain (HEI = 0%), [CH_4_] and [H_2_] both decrease due to reduction in fossil fuel emissions. As H_2_ emissions increase (HEI > 0), Δ[CH_4_] increases too. Up to the point that when HEI overcomes a critical threshold, there is an increase in atmospheric methane, i.e., Δ[CH_4_] > 0, even though methane emissions are lower. This critical HEI is in the range 8–10% for green H_2_ as it has a weak nonlinear dependence on the percentage of fossil fuel energy that is replaced by H_2_ (see also Supplementary Fig. 3).

The scenarios of blue H_2_ with 0.2% CH_4_ leak rates are not very different from the green H_2_ scenarios, with the critical HEI being in the range 7–8%. Regarding the scenarios of blue H_2_ with 1% CH_4_ leak rates, since there is basically no change in the methane emissions (Fig. 2b), the methane response is only associated with the reduction in OH availability due to the higher H_2_ concentration. The critical HEI is not defined for this blue H_2_ as the methane burden increases in all cases. The worst scenarios of blue H_2_ with 2% CH_4_ leak rates show drastic differences in the tropospheric concentrations of the two gases, which increase considerably, with a weakly nonlinear effect due to the drop in atmospheric OH.

The atmospheric methane response to future H_2_ production^2,33,34^ shows qualitatively similar results as a function of the H_2_ production pathway and the percentage of H_2_ lost to the atmosphere (Fig. 3b). Positive effects in terms of methane mitigation are observed only for green and blue H_2_ with low methane losses, if the H_2_ emission intensity is well below 10%. Otherwise, the tropospheric methane burden is enhanced.

We also evaluated the change in CO_2_ concentration (Δ[CO_2_e]) that would produce equivalent radiative forcing to the change in the equilibrium concentration of CH_4_ (Figs. 2c and 3b). We used the radiative efficiency of CH_4_ that includes indirect effects on O_3_ and stratospheric H_2_O^35^. Under the worst scenario of blue H_2_ production with 2% CH_4_ losses and 10% H_2_ losses, the rise in equilibrium CH_4_ due to future H_2_ production would be like adding 9 ppm of CO_2_ to the atmosphere (Fig. 3b). For the same blue H_2_, the rise in CH_4_ following the entire displacement of fossil fuels would be like adding around 70 ppm of CO_2_ (Fig. 2c). This is equivalent to around 50% of the CO_2_ increase from preindustrial times (278 ppm) to current days (417 ppm). Since the goal of keeping the global average temperature rise below 1.5 ^∘^C requires a mid-century maximum of CO_2_ close to 450 ppm, these results support previous concerns about the sustainability of blue H_2_^36^ unless fugitive emissions can be kept sufficiently low.

### Critical HEI for methane mitigation

The quantification of the critical hydrogen emission intensity (HEI_cr_) for methane mitigation is key to assess whether displacing fossil fuels with hydrogen would mitigate or enhance the tropospheric burden of CH_4_. Here we investigate how the HEI_cr_ is affected by the hydrogen production pathway and by two of the most uncertain terms in the CH_4_-H_2_-OH balance: (i) the partitioning of the OH sink among the tropospheric gases; (ii) the rate of H_2_ uptake by soil bacteria. The derivation of an analytical solution for the HEI_cr_ is reported in the “Methods”.

**Fig. 4:**
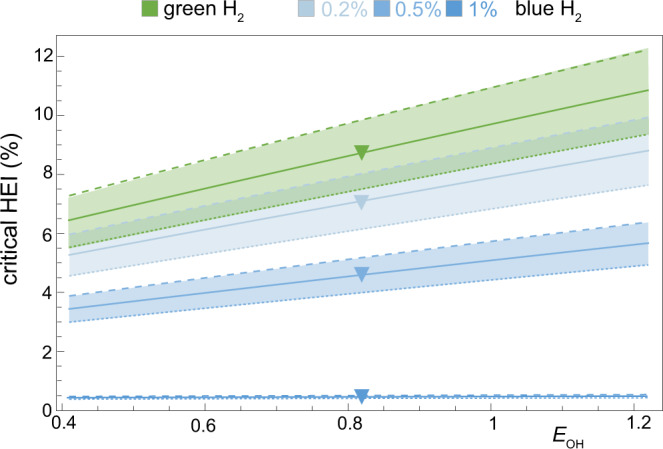
Critical hydrogen emission intensity (HEI) for methane mitigation. Critical HEI as a function of OH excess (*E*_OH_) and hydrogen production method (green and blue H_2_ with 0.2, 0.5, 1% CH_4_ leak rates, respectively). Dashed (dotted) lines are obtained for a 20% increase (decrease) in the H_2_ uptake rate by soil bacteria (*k*_*d*_). Triangles mark the critical HEI for the best estimate of *E*_OH_.

The very short lifetime of OH makes the quantification of its atmospheric dynamics extremely challenging. Indirect methods are typically used to estimate OH concentrations, sources, and sink partitioning^37–39^. Using a range of OH partition estimates^38,40^, we investigate the dependence of the HEI_cr_ to different values of OH excess (*E*_OH_), *E*_OH_ being the excess of OH that is consumed by other tropospheric gases besides hydrogen, methane, and carbon monoxide. Figure 4 shows the quasi-linear response of the HEI_cr_ to *E*_OH_. We stress that a variation in *E*_OH_ is equivalent to a variation in the OH sources since we preserve the current average OH concentration, which is relatively well constrained by inverse modeling^37,41^.

The HEI_cr_ is much lower for blue H_2_ than for green H_2_ because of the methane emissions associated with blue H_2_ production. For the current tropospheric conditions, we find that HEI_cr_ is around 9% for green H_2_, around 7% for blue H_2_ with 0.2% methane leak rates, and 4.5% for blue H_2_ with 0.5% methane leak rates. Blue H_2_ with 1% methane leak rate has a HEI_cr_ that is close to zero, as displacement of fossil fuel with this hydrogen does not reduce methane emissions (Fig. 3b). For even higher methane leak rates, the methane burden would increase regardless of the H_2_ emissions, so that the HEI_cr_ is negative.

The H_2_ uptake by soil bacteria is another crucial process in the evaluation of HEI_cr_ and in the overall CH_4_–H_2_–OH dynamics, since it accounts for 70–80% of H_2_ tropospheric removal^11^. Despite recent research on uptake modeling^42,43^ and the microbial characterization of the H_2_-oxidizing bacteria^44^, the spatial heterogeneity of the uptake as driven by local hydro-climatic and biotic conditions hinders bottom-up estimates of the global average uptake rate. In atmospheric studies, the average uptake rate is usually adjusted in order to obtain a reasonable simulation of observed surface hydrogen concentrations^14,45^. To account for these potential sources of uncertainties, we show how a ±20% variation in the uptake rate influences the critical HEI (bands in Fig. 4). A stronger biotic sink (dashed lines) reduces the consumption of OH by H_2_ and, consequently, increases the HEI_cr_. A weaker biotic sink (dotted line) has the opposite effect.

Regarding the impact of climate change on the H_2_ soil sink, recent studies indicate that increasing temperatures are expected to slightly favor the uptake on a global scale^14^, while shifts in rainfall regimes will be the significant drivers of H_2_ uptake changes at the local scale^43^. From a biotic perspective, the adaptability of H_2_-oxidizing bacteria to extreme environments^46^ suggests that their presence will remain widespread in the future, but their spatial heterogeneity may change as a result of climate and anthropogenic pressures.

Another source of uncertainties in the evaluation of HEI_cr_ is related to the estimate of CH_4_ emissions associated with fossil fuel use. Since there is a quasi linear relationship between these emissions and the HEI_cr_ (Eq. (16) in “Methods”), the same relative uncertainty of fossil fuel methane emissions (Fig. 1) applies to the HEI_cr_.

## Discussion

The success of the global net-zero transition hinges on hydrogen as a scalable low-carbon energy carrier that can replace fossil fuels in several hard-to-electrify energy and transport sectors. More than 20 governments and many companies have already announced strategies for hydrogen production, and the numbers are likely to increase as policy frameworks that facilitate hydrogen adoption are promoted^1,2^. Considerable investments are still needed to achieve such a transition, as the current hydrogen momentum falls short compared to net-zero goals. The Hydrogen Council^2^ estimates that there is a USD 540 billion gap between the investments of announced projects (USD 160 billion) on hydrogen production and the investments required by 2030 to be on a net-zero pathway (USD 700 billion).

While the positive effects of a more hydrogen-based economy are relatively established (e.g., lower CO_2_ emissions, decreased urban pollution, etc.), considerable uncertainty still surrounds the consequences of hydrogen emissions to the atmosphere, because of potential indirect GHG effects^14,19^. Here we have focused on the impact of a more hydrogen-based energy system on tropospheric methane, the second most important greenhouse gas.

We have shown how the replacement of fossil fuel energy with green or blue hydrogen could have very different consequences for tropospheric CH_4_, depending on the amount of hydrogen lost to the atmosphere and the methane emissions associated with hydrogen production (Figs. 2 and 3). Specifically, tropospheric CH_4_ would decrease due to the fossil fuel displacement only if the rate of H_2_ losses is kept below the critical HEI.

This is around 9 ± 3% for green H_2_ (Fig. 4). The same critical value would apply to other H_2_ colors that do not entail the use of fossil fuels, like white or orange H_2_ extracted from underground deposits^12,47^. The critical HEI for blue H_2_ is much lower due to the CH_4_ emission associated with blue H_2_ production. We have found that the methane emissions in a blue H_2_ economy could be higher than in a fossil fuel economy if the methane supply chain had an average leak rate above 1%. Furthermore, the superimposition of CH_4_ and H_2_ emissions may have undesired consequences for the tropospheric burden of CH_4_. This may be a potential problem in the near term, given that steam methane reforming will be used to bridge the gap between increasing H_2_ demand and limited green H_2_ production capacities^2^. Our results suggest that including hydrogen emissions would aggravate the greenhouse gas footprint of blue H_2_^36^.

In addition to the CH_4_ feedback, H_2_ emissions are also expected to impact ozone (O_3_) and stratospheric water vapor (H_2_O), with negative consequences for both air quality and radiative forcing. Accounting for these effects, we can provide a comparison between the radiative forcing of hydrogen-based and fossil fuel-based energy systems. Because both H_2_ and CH_4_ are short-lived gas compared to CO_2_, the time horizon for this comparison is crucial^48^. Here we consider 20-year and 100-year time horizons. The GWP of H_2_ is estimated at 11 ± 5 (100-year) and 33${}_{-13}^{+11}$ (20-year)^16^. The GWP of CH_4_ is estimated at 28 (100-year) and 80 (20-year)^35^. In an envisioned hydrogen economy that replaces the current fossil fuel industry, the H_2_ emissions could be in the range 23 to 370 Tg H_2_ yr^−1^, for a H_2_ emission intensity going from 1 to 10% (Fig. 2a). These emissions would have a radiative forcing impact of 0.7–12% (100-year) and 2–35% (20-year) of the current CO_2_ emissions from fossil fuels (≈35 Pg CO_2_ yr^−1^). If the global H_2_ economy relied on blue H_2_ with a 2% methane leakage rate, methane emissions would cause an additional radiative forcing impact that is around 10% (100-year) and 27% (20-year) of the current CO_2_ emissions from fossil fuels. Hence, in the worst scenario, up to 22% of the climate benefits of the hydrogen economy could be offset by gas losses over a 100-year horizon. The percentage could be as large as 65% over a 20-year horizon. These values could be higher on a regional scale if the leak rate of the natural gas supply chain is above 2%.

To maximize the climate benefit of hydrogen adoption, minimizing both H_2_ and CH_4_ losses across the supply chain of hydrogen production will need to be a priority. On the methane side, some governments and companies have already committed to reducing the leaks from the oil and gas sector, because this could be the most cost-effective and impactful action for near-term climate mitigation^21^. The International Energy Agency (IEA) estimates that, with the recent rise in natural gas prices, the abatement of methane emissions from the global gas and oil sector could be implemented at no net cost^49^. Hence, the accomplishment of this mitigation is only a matter of political will for the limited number of companies involved.

On the hydrogen side, the global value chain still has to be built. This offers the advantage of tackling the hydrogen emission problem ahead of time. On the one hand, energy companies will have a great interest in minimizing economic loss and safety risks due to hydrogen leaks. On the other hand, however, many technological challenges still need to be addressed. First, H_2_ containment may remain an issue even as technologies progress. The high diffusivity of the small H_2_ molecule has already challenged the scientific community’s ability to measure the H_2_ concentration in the atmosphere^50^ and in the firn air of ice sheets^51^. Second, while more field-based estimates of H_2_ losses are needed, there is currently no commercially available sensing technology able to detect small H_2_ leaks at the ppb level^48^. Third, global-space monitoring, which is bringing a much-needed transparency to the quantification of real methane emissions^27,31^, will also require new technology since H_2_, unlike CH_4_ or CO_2_, does not absorb infrared radiation. For all these reasons, the uncertainty about future emissions from the H_2_ value chain remains large.

Our versatile atmospheric model allowed a broad exploration of scenarios in a hydrogen-based energy system. Simulations with high resolution three-dimensional atmospheric chemistry models, which are more comprehensive but more computationally demanding, could refine our results for specific scenarios. In particular, a more detailed model could improve the assessment of H_2_ displacement of fossil fuels by accounting for the emission changes of other chemical species, like CO and NO_*x*_, which impact the CH_4_–H_2_–OH dynamics. Further analyses could also refine the potential changes in emission inventories due to H_2_ displacement of different fossil fuels.

## Methods

### The Model

With the increasing anthropogenic alteration of atmospheric chemistry, detailed three-dimensional atmospheric chemistry models have become critical to evaluate the atmospheric interactions with the climate forcing^52,53^. Nonetheless, thanks to their versatility, simplified models of atmospheric chemistry have also proven very useful to investigate the fundamental processes governing the coupling between atmospheric gases and the consequences of their possible perturbations (e.g., refs. 54–59). The insights obtained with the CH_4_–CO–OH model by Prather et al.^54^, in particular, led to a +40% revision of the IPCC’s GWP for CH_4_^60^. Here we extend Prather’s seminal model by adding the mass balance equation for atmospheric H_2_. The purpose is to identify the key components that control the H_2_ feedback on the tropospheric dynamics of CH_4_ (Fig. 1).

The chemical reactions considered are

1 $${{{{{{{{\rm{CH}}}}}}}}}_{4}+{{{{{{{\rm{OH}}}}}}}}\ \mathop{\longrightarrow }\limits^{{k}_{1}}\ \ldots \longrightarrow \alpha \,{{{{{{{{\rm{H}}}}}}}}}_{2}+{{{{{{{\rm{CO}}}}}}}}\ldots,\quad {R}_{{{{{{{{{\rm{CH}}}}}}}}}_{4}}={k}_{1}[{{{{{{{\rm{OH}}}}}}}}][{{{{{{{{\rm{CH}}}}}}}}}_{4}],$$

2 $${{{{{{{{\rm{H}}}}}}}}}_{2}+{{{{{{{\rm{OH}}}}}}}}\ \mathop{\longrightarrow }\limits^{{k}_{2}}\ \ldots,\quad {R}_{{{{{{{{{\rm{H}}}}}}}}}_{2}}={k}_{2}[{{{{{{{\rm{OH}}}}}}}}][{{{{{{{{\rm{H}}}}}}}}}_{2}],$$

3 $${{{{{{{\rm{CO}}}}}}}}+{{{{{{{\rm{OH}}}}}}}}\ \mathop{\longrightarrow }\limits^{{k}_{3}}\ \ldots,\quad {R}_{{{{{{{{\rm{CO}}}}}}}}}={k}_{3}[{{{{{{{\rm{OH}}}}}}}}][{{{{{{{\rm{CO}}}}}}}}],$$

4 $${{{{{{{\rm{X}}}}}}}}+{{{{{{{\rm{OH}}}}}}}}\ \mathop{\longrightarrow }\limits^{{k}_{4}}\ \ldots,\quad {R}_{{{{{{{{\rm{X}}}}}}}}}={k}_{4}[{{{{{{{\rm{OH}}}}}}}}][{{{{{{{\rm{X}}}}}}}}],$$

with *R* representing the rates of reactions, [ ⋅ ] the concentrations, and *k*_*i*_ the rate coefficients. We indicated only the products with which we are concerned, the CO and H_2_ produced by oxidation of CH_4_(1). H_2_ production through CH_4_ oxidation has yield *α* ≈ 0.37^13^. X encompasses all the other species, besides CH_4_, CO, and H_2_, that consume OH. Based on the above reactions, the balance equations for the CH_4_–H_2_–CO–OH system are

5 $$\frac{d[{{{{{{{{\rm{CH}}}}}}}}}_{4}]}{dt}={S}_{{{{{{{{{\rm{CH}}}}}}}}}_{4}}-{R}_{{{{{{{{{\rm{CH}}}}}}}}}_{4}}-{R}_{s},$$

6 $$\frac{d[{{{{{{{{\rm{H}}}}}}}}}_{2}]}{dt}={S}_{{{{{{{{{\rm{H}}}}}}}}}_{2}}+\alpha {R}_{{{{{{{{{\rm{CH}}}}}}}}}_{4}}-{R}_{{{{{{{{{\rm{H}}}}}}}}}_{2}}-{R}_{d},$$

7 $$\frac{d[{{{{{{{\rm{CO}}}}}}}}]}{dt}={S}_{{{{{{{{\rm{CO}}}}}}}}}+{R}_{{{{{{{{{\rm{CH}}}}}}}}}_{4}}-{R}_{{{{{{{{\rm{CO}}}}}}}}},$$

8 $$\frac{d[{{{{{{{\rm{OH}}}}}}}}]}{dt}={S}_{{{{{{{{\rm{OH}}}}}}}}}-{R}_{{{{{{{{{\rm{CH}}}}}}}}}_{4}}-{R}_{{{{{{{{{\rm{H}}}}}}}}}_{2}}-{R}_{{{{{{{{\rm{CO}}}}}}}}}-{R}_{{{{{{{{\rm{X}}}}}}}}},$$

where *R*_*d*_ = *k*_*d*_[H_2_] is the H_2_ uptake by soil bacteria, which plays a crucial role in the global balance of H_2_ since it accounts for around 70–80% of tropospheric removal^11,43,61^; *R*_*s*_ = *k*_*s*_[CH_4_] accounts for the smaller sinks of CH_4_, namely soil uptake, stratospheric loss and reactions with chlorine radicals^62^. For simplicity, we neglect the smaller sinks of H_2_, i.e., stratospheric loss (≈1% of removal^63^), and CO, i.e., soil uptake and stratospheric loss (<10% of removal^64^).

The solution at quasi steady state (i.e., d[ ⋅ ]/d*t* = 0) provides the sources for fixed tropospheric concentrations. Positive solutions for OH occurs if ${S}_{{{{{{{{\rm{OH}}}}}}}}} > (2+\alpha )({S}_{{{{{{{{{\rm{CH}}}}}}}}}_{4}}-{R}_{s})+{S}_{{{{{{{{\rm{CO}}}}}}}}}+{S}_{{{{{{{{{\rm{H}}}}}}}}}_{2}}-{R}_{d}$, i.e., when there is enough OH to oxidize all CO sources, the part of CH_4_ sources that is not balanced by smaller sinks, and the part of H_2_ sources that is not balanced by the soil uptake. The excess of OH consumed by other gases, besides CH_4_, CO, and H_2_, can be defined as ${E}_{{{{{{{{\rm{OH}}}}}}}}}={R}_{{{{{{{{\rm{X}}}}}}}}}/({R}_{{{{{{{{{\rm{CH}}}}}}}}}_{4}}+{R}_{{{{{{{{\rm{CO}}}}}}}}}+{R}_{{{{{{{{{\rm{H}}}}}}}}}_{2}})$. The values representing average tropospheric conditions are summarized in Table 1. The values of *S*_OH_ and *S*_CO_ are kept constant in all scenarios.

**Table 1:** Tropospheric budgets of key species and definition of linear stability modes

		CH_4_	H_2_	CO	OH	$-{\lambda }_{i}^{-1}$(yr)
Steady state	Concentration (ppb)	1890	530	80	10^6^ cm^−3^	
	Sources (ppb/yr)	226	265^a^	480^a^	1333	
	*τ* (yr)	8.3	2	0.17	1 s	
Linear stability	CH_4_ mode	1%	0.31%	0.64%	−0.39%	12.3
	H_2_ mode	−0.01%	1%	0.03%	−0.06%	2
	CO mode	−0.008%	0.001%	1%	−0.36%	0.2
	OH mode	...^b^	...^b^	...^b^	1%	1.5 s

^a^Sources for CO and H_2_ include production from CH_4_ oxidation.

^b^... is <10^−7^.

Sources are obtained from the system (5)–(8) at steady state with the current tropospheric concentrations. *τ* is the average lifetime of each gas. The modes are expressed as relative changes normalized so that the dominant species’ ratio is 1%. Reaction rates are defined as follows: *k*_1_ = 3.17 × 10^−15^ cm^3^/s; *k*_2_ = 3.8 × 10^−15^ cm^3^/s; *k*_3_ = 1.9 × 10^−13^ cm^3^/s; *k*_*s*_ = 0.02 yr^−1^; *k*_*d*_ = 0.38 yr^−1^ is such that soil uptake accounts for 75% of atmospheric H_2_ removal; *k*_4_[X] = 0.3 s^−1^ (*E*_OH_ = 0.82) is defined so that 45% of OH is consumed by the species X, 36% by CO, 14% by CH_4_, and 5% by H_2_^38^. Concentrations are converted to mixing ratios using 1 ppb = 1.57 × 10^10^ cm^−3^; sources are converted from ppb/yr to Tg/yr using 4.22 × 10^18^ kg as the troposphere mass^68^.

### Linear stability and transient dynamics

We investigate the effects of an emission pulse of H_2_ on the tropospheric system (5)–(8). The timescales and modes of the atmospheric response to chemical perturbations are defined by the eigenvalues and eigenvectors of the system^54,55^. Indicating with **c**(*t*) the solution vector of the system (5)–(8), the temporal dynamics of a small perturbation $\hat{{{{{{{{\bf{c}}}}}}}}}$ around **c** evolves as

9 $$\frac{{{{{{\rm{d}}}}}}\hat{{{{{{{{\bf{c}}}}}}}}}}{{{{{{\rm{d}}}}}}t}={{{{{{{\bf{J}}}}}}}}\hat{{{{{{{{\bf{c}}}}}}}}},$$

where **J** is the Jacobian of the system evaluated in **c**. For the equilibrium solution **c**_0_ representing the current tropospheric concentrations, the eigenvalues and eigenvectors, or modes, of the linearized system (9) are reported in Table 1. Since all eigenvalues are real and negative (*λ*_*i*_ < 0), the equilibrium solution **c**_0_ is a stable node. As a result, any small perturbation asymptotically decays in time with a timescale defined by the negative reciprocal of the eigenvalue.

Because the system equations are coupled, the decay timescale ($-{\lambda }_{i}^{-1}$) of a gas perturbation does not necessarily correspond to the gas steady state average lifetime (*τ*_*i*_). The CH_4_ perturbation, in particular, decays with a timescale that is much larger than what predicted by its steady state lifetime, i.e., $R\,=\,-{\lambda }_{{{{{{{{{\rm{CH}}}}}}}}}_{4}}^{-1}/{\tau }_{{{{{{{{{\rm{CH}}}}}}}}}_{4}} > 1$. This mechanism, known as the CH_4_ feedback effect^55,65^, has a crucial role in increasing the GWP and the environmental impact of CH_4_ emissions. Detailed models of atmospheric chemistry usually provide *R* around 1.3–1.4^65^. We find a marginally higher feedback factor, namely *R* ≈ 1.5, in agreement with previous findings using Prather’s box model^54,55,57^. The decay timescale of the H_2_ perturbation instead corresponds to the H_2_ average lifetime, namely $-{\lambda }_{{{{{{{{{\rm{H}}}}}}}}}_{2}}^{-1}\approx {\tau }_{{{{{{{{{\rm{H}}}}}}}}}_{2}}$, in agreement with results from detailed atmospheric chemistry models^19^.

While the modal eigenvalue analysis correctly captures the asymptotic stability of the solution **c**_0_, it does not describe the perturbation dynamics at finite times, i.e., before the asymptotic decay. Still within the domain of the linearized system (9), a more complete picture can be obtained by analyzing the temporal evolution of the solutions with specific attention to the emergence of transient growth phenomena, which are known to occur in systems where the modes are non-orthogonal, as in the present case. When large enough, a transient growth can even trigger nonlinearities that destabilize the equilibrium solution^66^.

**Fig. 5:**
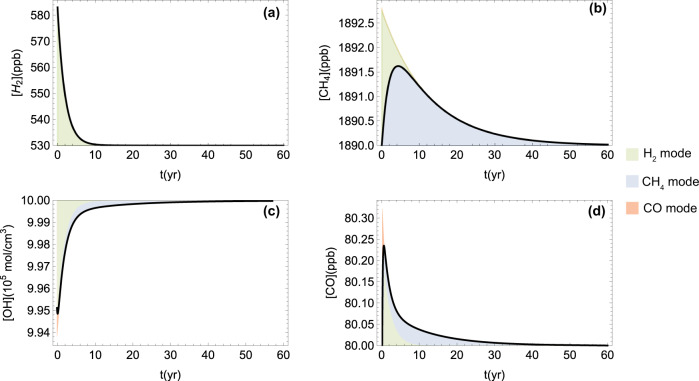
Transient dynamics. Tropospheric response to a pulse of H_2_ (10% increase of its concentration). Temporal dynamics of H_2_ (**a**), CH_4_ (**b**), OH (**c**), and CO (**d**). Colors highlight the contributions of the different modes. When different modes superimpose, the faster-decaying mode is shown on top of the others.

Figure 5 shows the transient growth phase of tropospheric CH_4_ and CO that follows a 10% perturbation of H_2_ concentration. Specifically, the pulse of H_2_ causes a drop in OH and a build-up of CH_4_ that lasts a few years, while the H_2_ perturbation decays with the timescale ${\tau }_{{{{{{{{{\rm{H}}}}}}}}}_{2}}$. The CH_4_ build-up then decays in the same manner as would a direct pulse of CH_4_ with a timescale defined by the CH_4_ feedback effect. In analytical terms, the perturbation of tropospheric CH_4_ mainly due to the excitation of H_2_ and CH_4_ modes is given by $\delta [{{{{{{{{\rm{CH}}}}}}}}}_{4}]\approx 2.76{{{{{\rm{e}}}}}}^{{\lambda }_{{{{{{{{{\rm{CH}}}}}}}}}_{4}}t}-2.82{{{{{\rm{e}}}}}}^{{\lambda }_{{{{{{{{{\rm{H}}}}}}}}}_{2}}t}+0.06{{{{{\rm{e}}}}}}^{{\lambda }_{{{{{{{{\rm{CO}}}}}}}}}t}$.

Using this result in traditional GWP formulas^35^ yields a GWP for H_2_ due to direct CH_4_ perturbation around 7.8 with the 100-year time-horizon and 22 with the 20-year time horizon. It is estimated that around half of the H_2_ indirect radiative forcing is due to the direct CH_4_ perturbation, and the other half to the O_3_ and stratospheric H_2_O impacts caused by both H_2_ and H_2_-induced CH_4_ perturbations^14^. Taking this into account yields a total GWP for H_2_ of 15.6 with the 100-year time-horizon and 44 with the 20-year time-horizon. These values are in the upper range of the recent estimates of 11 ± 5 for GWP100 and 33${}_{-13}^{+11}$ for GWP20 obtained with a detailed model of atmospheric chemistry^16^. Notably, the consequences of the H_2_ pulse on CH_4_ are relatively small in magnitude because most of the additional H_2_ is oxidized by soil bacteria and not by OH. The stability of this biotic sink as affected by climate change and anthropic pressure is hence a crucial aspect for the impact of future H_2_ emissions, as further discussed in the main text.

### Critical hydrogen emission intensity

We here derive an explicit expression for the critical H_2_ emission intensity (HEI_cr_) for methane mitigation, defined as the emission rate that offsets the H_2_ replacement of fossil fuels. The expression is derived for an infinitesimal replacement of fossil fuel energy with H_2_ (d*E* in ExJ/yr), but well approximates the critical HEI for finite replacement of fossil fuel energy (see Supplementary Fig. 3). As a first step, we differentiate the system (5)–(8) at equilibrium (d[⋅]/d*t* = 0) with respect to *E*. This yields

10 $${S}_{{{{{{{{{\rm{CH}}}}}}}}}_{4},E}-{k}_{1}[{{{{{{{{\rm{CH}}}}}}}}}_{4}]{[{{{{{{{\rm{OH}}}}}}}}]}_{E}=0,$$

11 $${S}_{{{{{{{{{\rm{H}}}}}}}}}_{2},E}+\alpha {k}_{1}[{{{{{{{{\rm{CH}}}}}}}}}_{4}]{[{{{{{{{\rm{OH}}}}}}}}]}_{E}-{k}_{2}{\left([{{{{{{{{\rm{H}}}}}}}}}_{2}][{{{{{{{\rm{OH}}}}}}}}]\right)}_{E}-{k}_{d}{[{{{{{{{{\rm{H}}}}}}}}}_{2}]}_{E}=0,$$

12 $${k}_{1}[{{{{{{{{\rm{CH}}}}}}}}}_{4}]{[{{{{{{{\rm{OH}}}}}}}}]}_{E}-{k}_{3}{\left([{{{{{{{\rm{CO}}}}}}}}][{{{{{{{\rm{OH}}}}}}}}]\right)}_{E}=0,$$

13 $${k}_{1}[{{{{{{{{\rm{CH}}}}}}}}}_{4}]{[{{{{{{{\rm{OH}}}}}}}}]}_{E}+{k}_{2}{\left([{{{{{{{{\rm{H}}}}}}}}}_{2}][{{{{{{{\rm{OH}}}}}}}}]\right)}_{E}+{k}_{3}{\left([{{{{{{{\rm{CO}}}}}}}}][{{{{{{{\rm{OH}}}}}}}}]\right)}_{E}+{k}_{4}[{{{{{{{\rm{X}}}}}}}}]{[{{{{{{{\rm{OH}}}}}}}}]}_{E}=0,$$

where subscript *E* indicates d ⋅ /d*E*. ${[{{{{{{{{\rm{CH}}}}}}}}}_{4}]}_{E}\,=\,0$ because of the definition of the critical H_2_ emission intensity, which leaves the methane concentration unaltered. We consider that only H_2_ and CH_4_ sources vary with *E*, while *S*_OH,*E*_ = *S*_CO,*E*_ = 0. These variations can be estimated as

14 $${S}_{{{{{{{{{\rm{H}}}}}}}}}_{2},E}={a}_{{{{{{{{{\rm{H}}}}}}}}}_{2}}\left(-{{{{{{{{\rm{ff}}}}}}}}}_{{{{{{{{{\rm{H}}}}}}}}}_{2}}+\frac{{{{{{{{\rm{HEI}}}}}}}}}{{\eta }_{{{{{{{{{\rm{H}}}}}}}}}_{2}}(1-{{{{{{{\rm{HEI}}}}}}}})}\right),$$

15 $${S}_{{{{{{{{{\rm{CH}}}}}}}}}_{4},E}={a}_{{{{{{{{{\rm{CH}}}}}}}}}_{4}}\left(-{{{{{{{{\rm{ff}}}}}}}}}_{{{{{{{{{\rm{CH}}}}}}}}}_{4}}+\frac{r\,{{{{{{{\rm{MEI}}}}}}}}}{{\eta }_{{{{{{{{{\rm{H}}}}}}}}}_{2}}(1-{{{{{{{\rm{HEI}}}}}}}})}\right),$$

where HEI and MEI are the hydrogen and methane emission intensities, respectively (MEI = 0 for green H_2_); ${\eta }_{{{{{{{{{\rm{H}}}}}}}}}_{2}}$ is H_2_ higher heating value; *r* is the amount of CH_4_ needed to produce a unit of blue H_2_; ${{{{{{{{\rm{ff}}}}}}}}}_{{{{{{{{{\rm{CH}}}}}}}}}_{4}}$ and ${{{{{{{{\rm{ff}}}}}}}}}_{{{{{{{{{\rm{H}}}}}}}}}_{2}}$ are the average amounts of CH_4_ and H_2_ emitted per ExJ of fossil fuel energy; ${a}_{{{{{{{{{\rm{H}}}}}}}}}_{2}}$ and ${a}_{{{{{{{{{\rm{CH}}}}}}}}}_{4}}$ are conversion factors.

Substituting Eqs. (14), (15) into the system (10)–(13) and after some algebra, one obtains the critical H_2_ emission intensity

16 $${{{{{{{{\rm{HEI}}}}}}}}}_{{{{{{{{\rm{cr}}}}}}}}}=\frac{A\left({{{{{{{{\rm{ff}}}}}}}}}_{{{{{{{{{\rm{CH}}}}}}}}}_{4}}\,{\eta }_{{{{{{{{{\rm{H}}}}}}}}}_{2}}-r\,{{{{{{{\rm{MEI}}}}}}}}\right)+B\,{{{{{{{{\rm{ff}}}}}}}}}_{{{{{{{{{\rm{H}}}}}}}}}_{2}}\,{\eta }_{{{{{{{{{\rm{H}}}}}}}}}_{2}}}{A\,{{{{{{{{\rm{ff}}}}}}}}}_{{{{{{{{{\rm{CH}}}}}}}}}_{4}}\,{\eta }_{{{{{{{{{\rm{H}}}}}}}}}_{2}}+B\left({{{{{{{{\rm{ff}}}}}}}}}_{{{{{{{{{\rm{H}}}}}}}}}_{2}}{\eta }_{{{{{{{{{\rm{H}}}}}}}}}_{2}}+1\right)}.$$

where the dependence to the atmospheric composition is embedded in $A={k}_{d}({k}_{4}[{{{{{{{\rm{X}}}}}}}}]+{k}_{2}[{{{{{{{{\rm{H}}}}}}}}}_{2}]+2{k}_{1}[{{{{{{{{\rm{CH}}}}}}}}}_{4}])+{k}_{2}[{{{{{{{\rm{OH}}}}}}}}]\left((\alpha+2){k}_{1}[{{{{{{{{\rm{CH}}}}}}}}}_{4}]+{k}_{4}[{{{{{{{\rm{X}}}}}}}}]\right)$ and *B* = 8*k*_1_*k*_2_[CH_4_][OH]. Parameters have been defined as follows: ${\eta }_{{{{{{{{{\rm{H}}}}}}}}}_{2}}=0.143$ ExJ/Tg${}_{{{{{{{{{\rm{H}}}}}}}}}_{2}}$, *r* = 3.2 kg${}_{{{{{{{{{\rm{CH}}}}}}}}}_{4}}$/kg${}_{{{{{{{{{\rm{H}}}}}}}}}_{2}}$, ${{{{{{{{\rm{ff}}}}}}}}}_{{{{{{{{{\rm{CH}}}}}}}}}_{4}}=0.225$ Tg${}_{{{{{{{{{\rm{CH}}}}}}}}}_{4}}$/ExJ, ${{{{{{{{\rm{ff}}}}}}}}}_{{{{{{{{{\rm{H}}}}}}}}}_{2}}=0.0225$ Tg${}_{{{{{{{{{\rm{H}}}}}}}}}_{2}}$/ExJ, ${a}_{{{{{{{{{\rm{CH}}}}}}}}}_{4}}\,=\,0.43$ ppb/Tg, ${a}_{{{{{{{{{\rm{H}}}}}}}}}_{2}}\,=\,8{a}_{{{{{{{{{\rm{CH}}}}}}}}}_{4}}$. To obtain the value of *r*, we used the estimate of 3.7 kg of natural gas for kg of H_2_^67^, which includes feedstock and energy requirements, and we assumed that 85% of natural gas by weight is composed by methane. ${{{{{{{{\rm{ff}}}}}}}}}_{{{{{{{{{\rm{CH}}}}}}}}}_{4}}$ and ${{{{{{{{\rm{ff}}}}}}}}}_{{{{{{{{{\rm{H}}}}}}}}}_{2}}$ are obtained as the ratio between the global CH_4_ and H_2_ emissions due to fossil fuel use and the global fossil fuel energy.

## Supplementary information

Supplementary Information

Peer Review File

Description of Additional Supplementary Files

Supplementary Dataset 1

## Data Availability

All data generated during this study are provided in the supplementary dataset file.

## Code availability

The code used to generate the results is provided in the supplementary dataset file.
