# Supplementary material for: Risk of the hydrogen economy for atmospheric methane
Source: Nat Commun. 2022 Dec 13;13:7706. doi: 10.1038/s41467-022-35419-7 (PMC9747913; doi:10.1038/s41467-022-35419-7)
Supplement: Supplementary file 1 — Supplementary Information [file 41467_2022_35419_MOESM1_ESM.pdf]

Supplementary Information for 'Risk of The Hydrogen Economy For Atmospheric Methane'

Matteo B. Bertagni, Stephen Pacala, Fabien Paulot and Amilcare Porporato

*Corresponding author(s). E-mail(s): matteobb@princeton.edu;

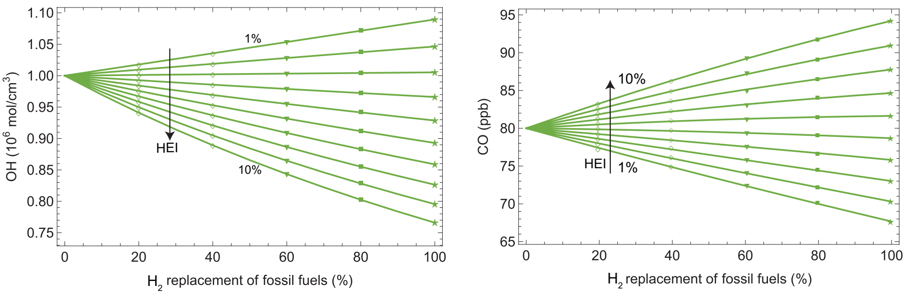

Supplementary Figure 1 Steady state concentrations of OH and CO. Equilibrium [OH] and [CO] vs the percentage of fossil fuel replacement by green H_2. Scenarios of Fig. 2 in the main text. HEI is the hydrogen emission intensity.

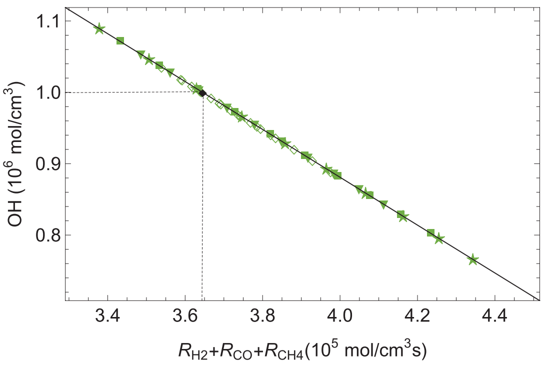

Supplementary Figure 2 Relationship between OH concentration and sinks. [OH] vs the sum of CH_4, CO and H_2 tropospheric sinks for the green H_2 scenarios of Fig. 2 in the main text. We find a linear relationship since $R_{CH_4} + R_{CO} + R_{H2} = S_{OH} - k_4[X][OH]$ from eq. (8) in the main text. More detailed atmospheric chemistry models sometimes find a nonlinear scaling with exponent -3/2, but with large inter and intramodel variability (see Ref. [39] in the main text).

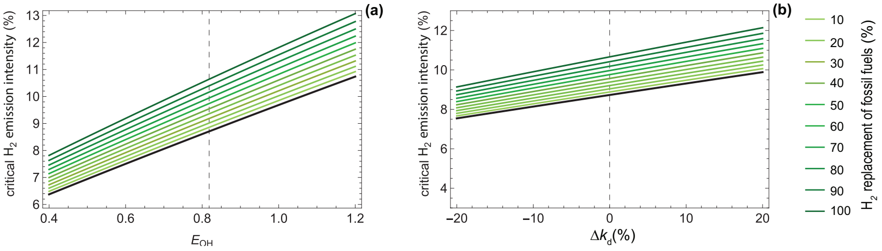

Supplementary Figure 3 Critical H_2 emission intensity (HEI). Critical HEI of green H_2 for methane mitigation as a function of (a) the OH excess (E_{OH}) and (b) the rate of H_2 soil uptake. Black lines are HEI_{cr} from eq. (16) in the main text. Colored lines are obtained numerically for a finite replacement of fossil fuel energy with green H_2.
