# Supplementary material for: Risk of the hydrogen economy for atmospheric methane
Source: Nat Commun. 2022 Dec 13;13:7706. doi: 10.1038/s41467-022-35419-7 (PMC9747913; doi:10.1038/s41467-022-35419-7)
Supplement: Supplementary file 3 — Description of Additional Supplementary Files [file 41467_2022_35419_MOESM3_ESM.pdf]

Description of Additional Supplementary Files:

Supplementary Dataset 1: Mathematica notebook with the minimalist atmospheric model. The notebook contains all analyses, results, and figures presented in the manuscript.
